# Supplementary material for: Replicated blood-based biomarkers for myalgic encephalomyelitis not explicable by inactivity
Source: EMBO Mol Med. 2025 Jun 20;17(7):1868–91. doi: 10.1038/s44321-025-00258-8 (PMC12254397; doi:10.1038/s44321-025-00258-8)
Supplement: Supplementary file 16 — Expanded View Figures [file 44321_2025_258_MOESM16_ESM.pdf]

# Expanded View Figures

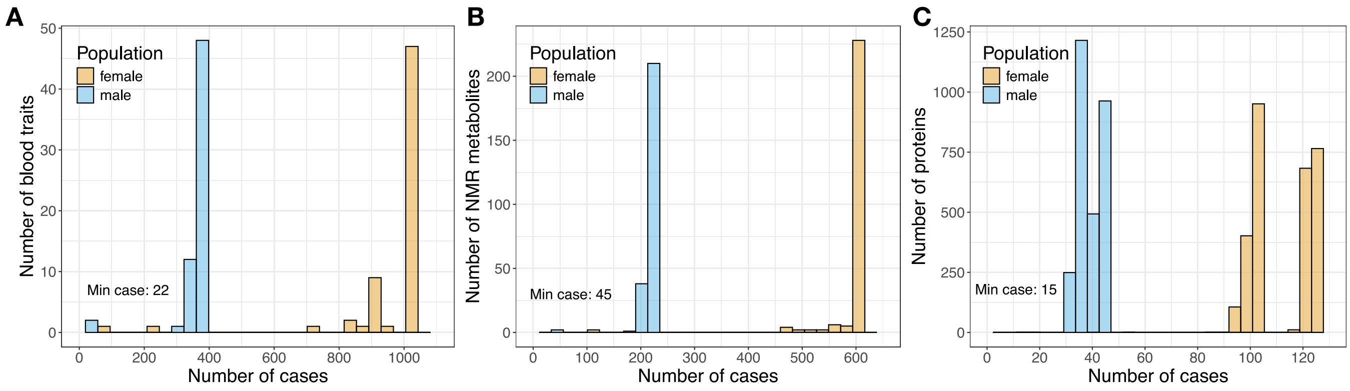


**Figure EV1. ME/CFS sample sizes for males and females, restricting to complete cases (individuals for whom a measurement is available).**

The minimum number of cases is indicated on each plot. (**A**) Blood traits, (**B**) NMR metabolites, (**C**) Proteomics. Neither of the two proteins with case sample size below 30 is significant after FDR correction. Full sample size data is provided as Dataset EV6.

 © The Author(s)

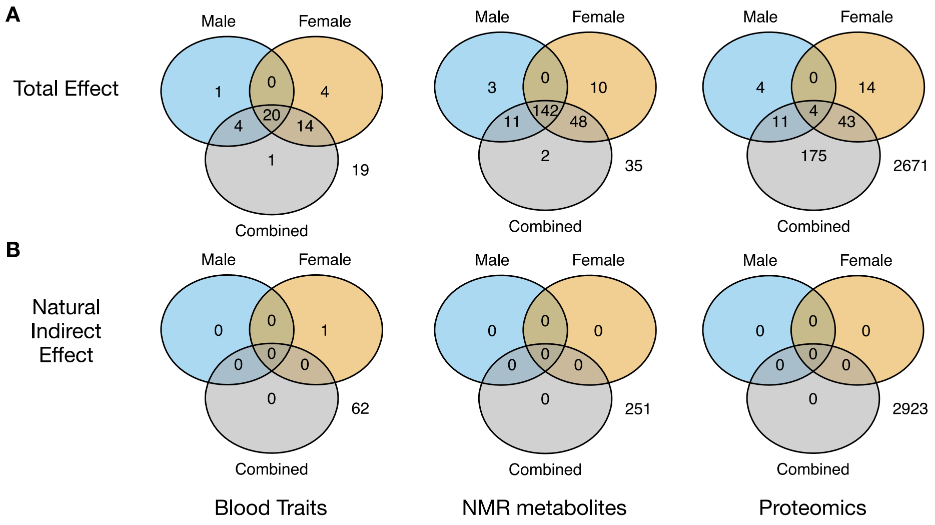


**Figure EV2. Overview of significant associational total effects and natural indirect effects (NIE) in the male, female, and combined populations.**

Venn diagrams displaying the number of significant findings in the males, females, combined and their intersection, mediator 874, for (**A**) total effect, and (**B**) NIE.

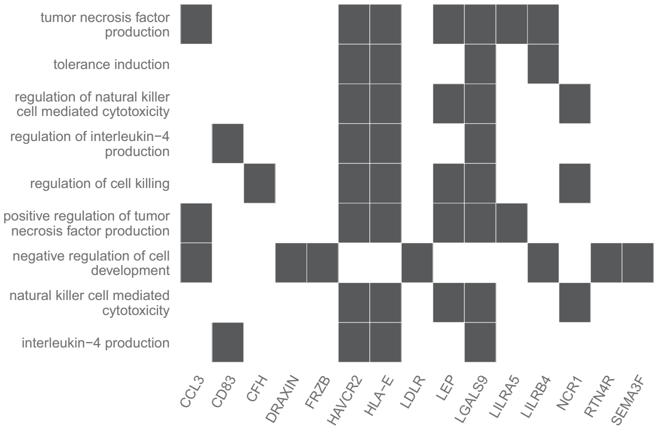


**Figure EV3. GO pathway enrichment (Ashburner et al, 2000) for proteins with a significant positive total effect for ME/CFS vs control, restricted to females only.**

This is the subset with maximal power for GO analysis. All effects are TE, i.e., there are no significant NIE for proteins. We performed a similar pathway GO enrichment analysis for proteins with a significant positive total effect for ME/CFS vs control on the population of males and the combined dataset, as well as all significant negative total effects and all significant total effects on the female, male and combined populations. These resulted in no significant GO term enrichments at FDR <0.05. All measured UKB proteins were used as background for the GO analyses.

 © The Author(s)

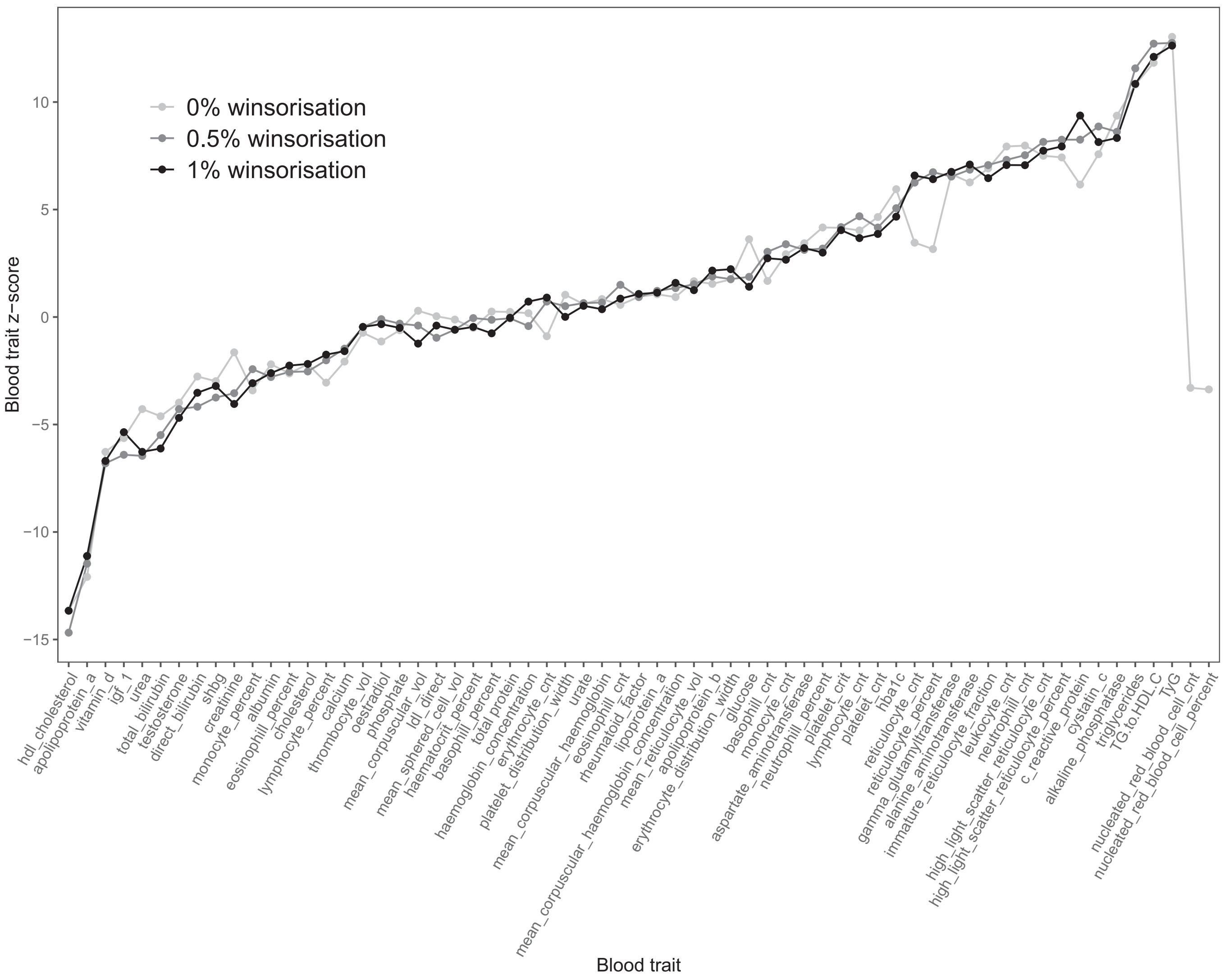


**Figure EV4. Significant blood traits are robust to winsorisation.**

The points represent total effect z-scores for blood traits in the combined female and male analysis. The three shades of grey represent different degrees of winsorisation of the original data, with cases and controls combined prior to winsorisation. Nucleated red blood cell count and percent are only estimable at 0% winsorisation because for 0.5% winsorisation the number of cases is ≤5. Fib4 and eGFR composite measures were not estimated for 0% winsorisation due to extreme values in control samples (e.g., individuals with platelet counts close to 0). Full results and sample sizes can be found in Dataset EV9.

© The Author(s)

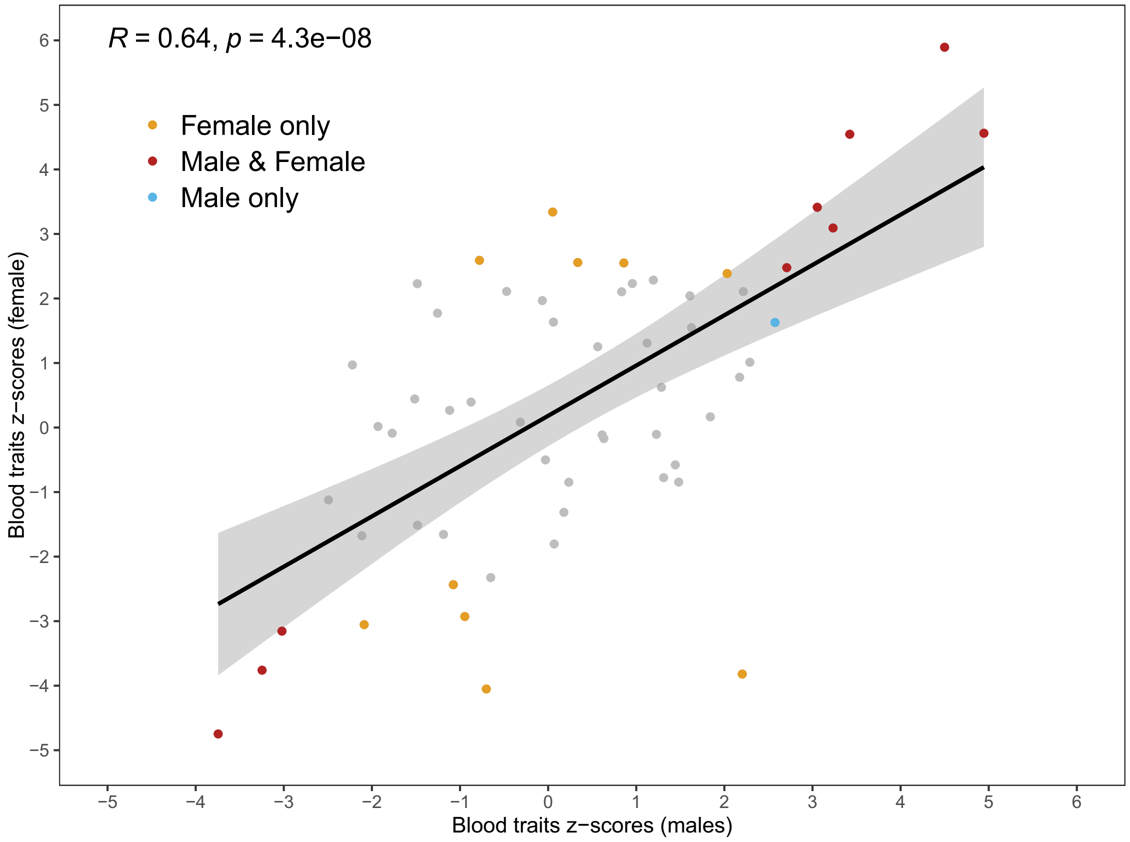


**Figure EV5. NDE of ME/CFS on blood traits for females and males, for mediator 874, with BMI included as a confounding variable.**

The Pearson correlation is 0.64 and significant ($P = 4.3 \times 10^{-8}$). Data points relating to nucleated red blood cells are not shown due to >90% data missingness. Full results and sample sizes can be found in Dataset EV13.

 © The Author(s)
